# Author Correction: Long-term carbon sink in Borneo’s forests halted by drought and vulnerable to edge effects

**DOI:** 10.1038/s41467-018-02920-x

**Published:** 2018-01-19

**Authors:** Lan Qie, Simon L. Lewis, Martin J. P. Sullivan, Gabriela Lopez-Gonzalez, Georgia C. Pickavance, Terry Sunderland, Peter Ashton, Wannes Hubau, Kamariah Abu Salim, Shin-Ichiro Aiba, Lindsay F. Banin, Nicholas Berry, Francis Q. Brearley, David F. R. P. Burslem, Martin Dančák, Stuart J. Davies, Gabriella Fredriksson, Keith C. Hamer, Radim Hédl, Lip Khoon Kho, Kanehiro Kitayama, Haruni Krisnawati, Stanislav Lhota, Yadvinder Malhi, Colin Maycock, Faizah Metali, Edi Mirmanto, Laszlo Nagy, Reuben Nilus, Robert Ong, Colin A. Pendry, Axel Dalberg Poulsen, Richard B. Primack, Ervan Rutishauser, Ismayadi Samsoedin, Bernaulus Saragih, Plinio Sist, J. W. Ferry Slik, Rahayu Sukmaria Sukri, Martin Svátek, Sylvester Tan, Aiyen Tjoa, Mark van Nieuwstadt, Ronald R. E. Vernimmen, Ishak Yassir, Petra Susan Kidd, Muhammad Fitriadi, Nur Khalish Hafizhah Ideris, Rafizah Mat Serudin, Layla Syaznie Abdullah Lim, Muhammad Shahruney Saparudin, Oliver L. Phillips

**Affiliations:** 1 School of Geography, University of Leeds, LS2 9JT Leeds, UK; 2 Department of Life Sciences, Imperial College London, Silwood Park Campus, SL5 7PY Ascot, UK; 3 Department of Geography, University College London, WC1E 6BT London, UK; 4 Center for International Forestry Research, Jl. CIFOR, Situ Gede, 16115 Bogor (Barat), Indonesia; 5 School of Environmental and Marine Science, James Cook University, 1 James Cook Dr, Townsville City, QLD 4811 Australia; 6 Department of Organismic and Evolutionary Biology, Harvard University, 22 Divinity Avenue, Cambridge, MA 02138 USA; 7 Laboratory for wood Biology and Xylarium, Royal Museum for Central Africa, Leuvensesteenweg 13, 3080 Tervuren, Belgium; 8 Environmental and Life Sciences Programme, Faculty of Science, Universiti Brunei Darussalam, Jalan Tungku Link, BE1410 Gadong, Brunei Darussalam; 9 Graduate School of Science and Engineering, Kagoshima University, 890-0065 Kagoshima, Japan; 10 Centre for Ecology and Hydrology, EH26 0QB Penicuik, UK; 11 Bioclimate, Thorn House, 5 Rose Street, EH2 2PR Edinburgh, UK; 12 School of Science and the Environment, Manchester Metropolitan University, Chester Street, M1 5GD Manchester, UK; 13 School of Biological Sciences, University of Aberdeen, Cruickshank Building, St Machar Drive, AB24 3UU Aberdeen, UK; 14 Department of Ecology & Environmental Sciences, Faculty of Science, Palacký University in Olomouc, Šlechtitelů 27, CZ-78371 Olomouc, Czech Republic; 15 Center for Tropical Forest Science-Forest Global Earth Observatory, Smithsonian Tropical Research Institute, Washington, DC 20013 USA; 16 Asian School of the Environment, Nanyang Technological University, 50 Nanyang Avenue, 639798 Singapore, Singapore; 17 Institute for Biodiversity and Ecosystem Dynamics, University of Amsterdam, 1012 WX Amsterdam, The Netherlands; 18 Pro Natura Foundation, Jl. Jend. Sudirman No. 37, 76112 Balikpapan, Indonesia; 19 Pan Eco, SOCP, Jl. Wahid Hasyim No. 51/74, 20154 Medan, Indonesia; 20 School of Biology, University of Leeds, LS2 9JT Leeds, UK; 21 Department of Vegetation Ecology, Institute of Botany, The Czech Academy of Sciences, Lidicka 25/27, CZ-60200 Brno, Czech Republic; 22 Department of Botany, Faculty of Science, Palacký University in Olomouc, Šlechtitelů 27, CZ-78371 Olomouc, Czech Republic; 23 Tropical Peat Research Institute, Biological Research Division, Malaysian Palm Oil Board, Bandar Baru Bangi, 43000 Kajang, Malaysia; 24 Graduate School of Agriculture, Kyoto University, 606-8502 Kyoto, Japan; 25 Forest Research and Development Center, Research, Development and Innovation Agency, Ministry of Environment and Forestry, Jl. Gunung Batu No 5, 16610 Bogor, Indonesia; 26 Department of Animal Science and Food Processing, Faculty of Tropical Agrisciences, Czech University of Life Sciences, Kamýcká 129, 165 00 Praha 6 – Suchdol, Prague, Czech Republic; 27 Ústí nad Labem Zoo, Drážďanská 23, 400 07 Ústí nad Labem, Czech Republic; 28 Environmental Change Institute, School of Geography and the Environment, University of Oxford, OX1 3QY Oxford, UK; 29 International Tropical Forestry, Faculty of Science and Natural Resources, Universiti Malaysia Sabah, Jl. UMS, 88400 Kota Kinabalu, Malaysia; 30 Research Center for Biology, Indonesian Institute of Sciences, Jl. Raya Jakarta-Bogor KM 46, 16911 Cibinong, Indonesia; 31 Universidade Estadual de Campinas, 13083-970 Campinas, Brazil; 32 Sabah Forestry Department Forest Research Centre, Mile 14 Jl. Sepilok, 90000 Sandakan, Malaysia; 33 Royal Botanic Garden Edinburgh, EH3 5LR Edinburgh, UK; 34 Biology Department, Boston University, 5 Cummington Mall, Boston, MA 02215 USA; 35 Smithsonian Tropical Research Institute, Balboa, Ancon, 03092 Panama; 36 Carboforexpert, 1248 Hermance, Switzerland; 37 Faculty of Forestry, Mulawarman University, Jl. Pasir Balengkong, 75123 Samarinda, Indonesia; 38 Forests and Societies Research Unit, CIRAD-Univ. Montpellier, Campus International de Baillarguet, TA C-105/D, 34398 Montpellier Cedex 5, France; 39 Department of Forest Botany, Dendrology and Geobiocoenology, Faculty of Forestry and Wood Technology, Mendel University in Brno, Zemedelska 3, 613 00 Brno, Czech Republic; 40 CTFS-ForestGEO Program, Lambir, Miri, Sarawak 98000 Malaysia; 41 Agriculture Faculty of Tadulako University, Jln Soekarno Hatta km 09, 94118 Tondo, Indonesia; 42 Utrecht University, Domplein 29, 3512 JE Utrecht, The Netherlands; 43 Deltares, Boussinesqweg 1, 2629 HV Delft, The Netherlands; 44 Balitek-KSDA, Research, Development and Innovation Agency, Ministry of Environment and Forestry, Jl. Soekarno Hatta KM. 38, RT 09 Samboja, Indonesia; 45 Instituto de Investigaciones Agrobiológicas de Galicia (IIAG), Consejo Superior de Investigaciones Científicas (CSIC), Santiago de Compostela, 15705 Spain; 46 Sungai Wain Protected Forest Management Unit, KM. 23, Kel. Karang Joang, 76101 Balikpapan, Indonesia

Correction to: *Nature Communications* 10.1038/s41467-017-01997-0, Article published online 19 December 2017

The original version of this Article contained an error in the third sentence of the abstract and incorrectly read “Here, using long-term plot monitoring records of up to half a century, we find that intact forests in Borneo gained 0.43 Mg C ha^−1^ year^−1^ (95% CI 0.14–0.72, mean period 1988–2010) above-ground live biomass”, rather than the correct “Here, using long-term plot monitoring records of up to half a century, we find that intact forests in Borneo gained 0.43 Mg C ha^−1^ year^−1^ (95% CI 0.14–0.72, mean period 1988–2010) in above-ground live biomass carbon”. This has now been corrected in both the PDF and HTML versions of the Article.

