# Supplementary material for: Long-term carbon sink in Borneo’s forests halted by drought and vulnerable to edge effects
Source: Nat Commun. 2017 Dec 19;8:1966. doi: 10.1038/s41467-017-01997-0 (PMC5736600; doi:10.1038/s41467-017-01997-0)
Supplement: Supplementary file 1 — Supplementary Information [file 41467_2017_1997_MOESM1_ESM.pdf]

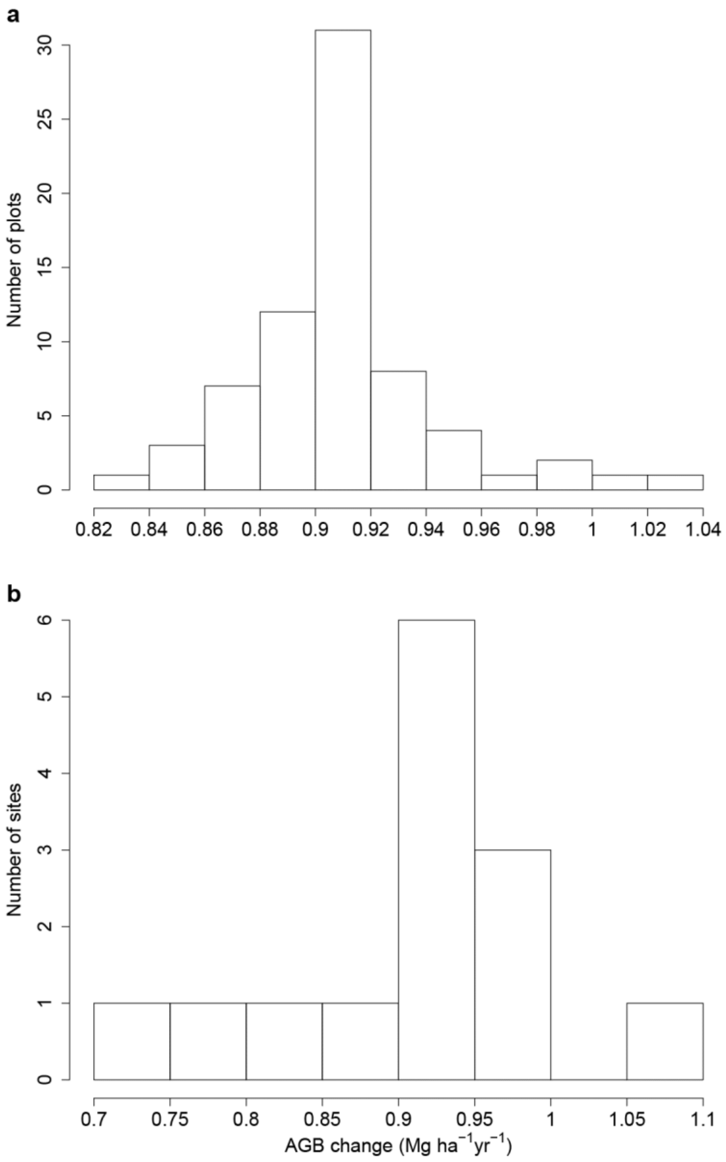

Supplementary Figure 1. The effect of individual plots and sites on the linear mixed effects (LME) model estimate for forest interior sink using omission tests. This was computed by omitting a single plot (**a**) or site (**b**) each time and estimating above-ground live biomass (AGB, dry weight) change in forest interior as was done in the main analysis using all plots, where forest interior AGB change was estimated at 0.91 Mg ha^{-1} yr^{-1}. The left tails represent plots/sites with outlying high AGB gains (thus when omitted resulted in lower estimates), and the right tails represent outlying low AGB gains (i.e., high AGB losses, thus when omitted resulted in higher estimates).

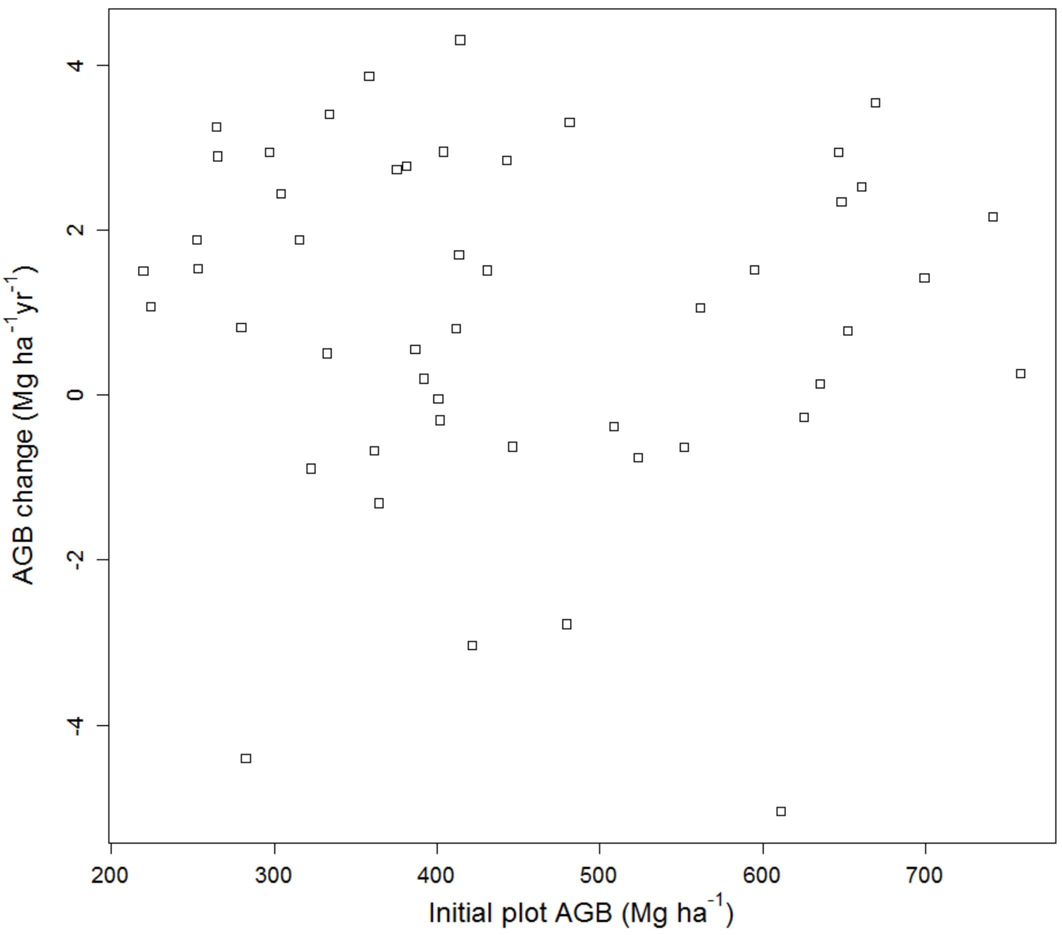

Supplementary Figure 2. Relationships between plot above-ground live biomass (AGB, dry mass) change and initial plot AGB for intact interior forest plots. The disturbance-recovery hypothesis predicts that forests at earlier successional stages (corresponding to lower AGB values) will show greater AGB gains. In Borneo's intact interior forests (n=49), plot AGB change was not correlated with plot AGB.

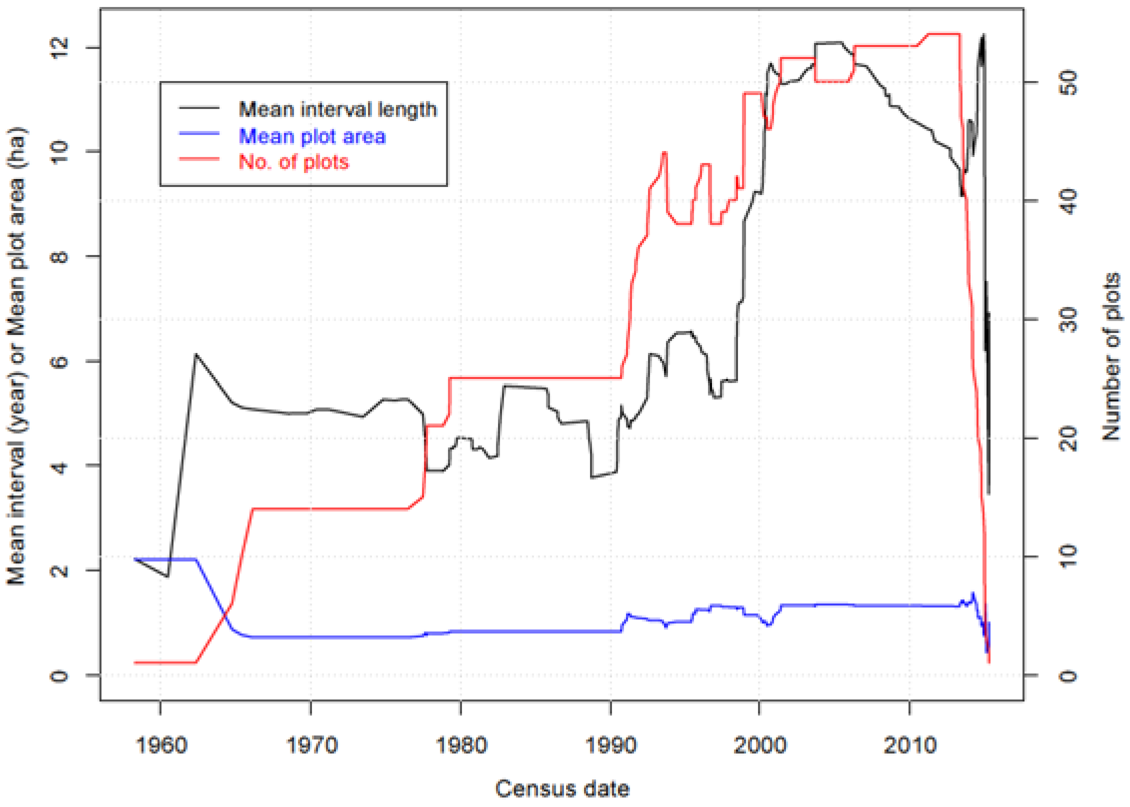

Supplementary Figure 3. Mean interval length, mean plot area and number of plots included in this study over the period 1958 to 2015.

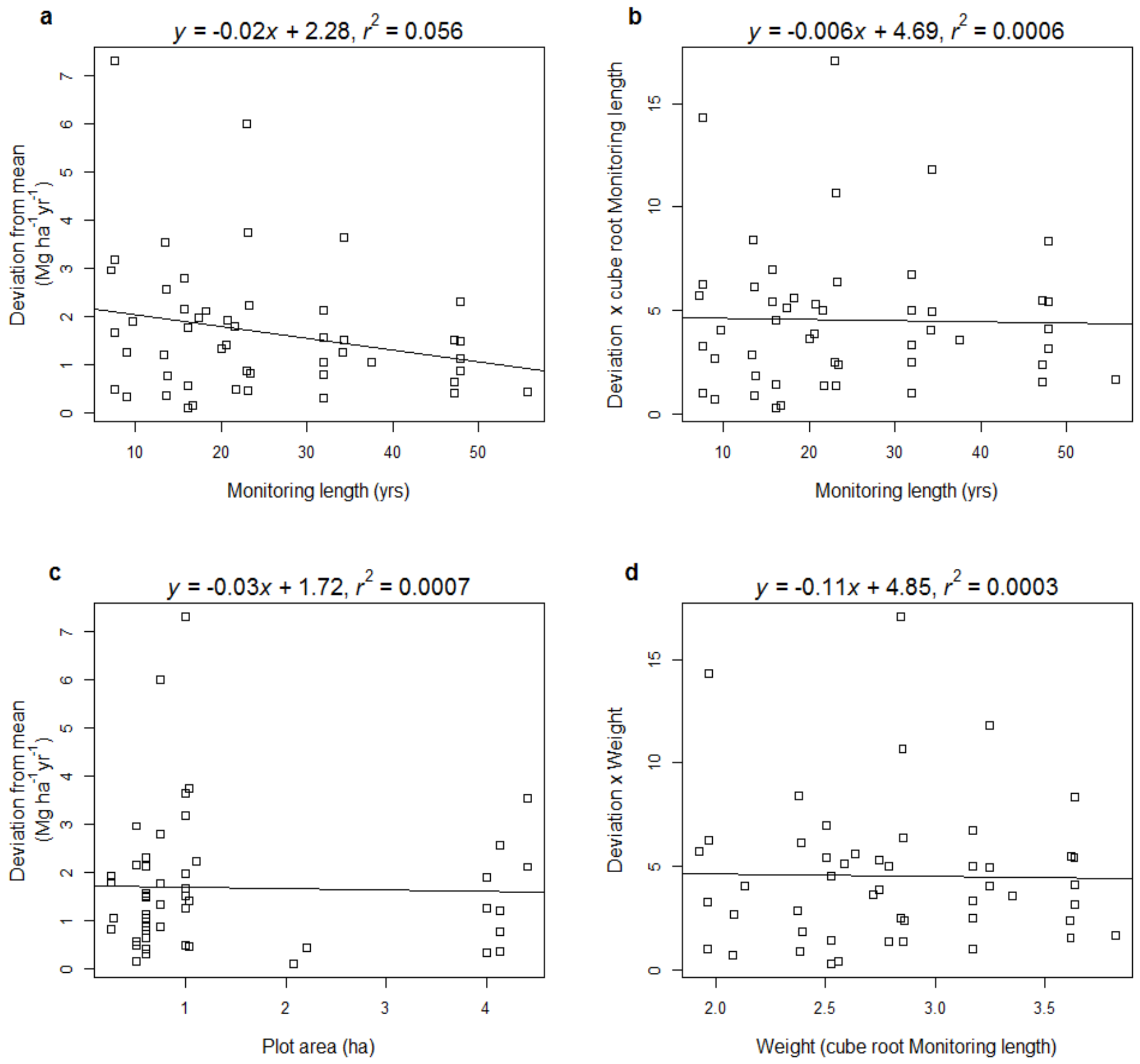

Supplementary Figure 4. Determining appropriate weight for individual plot above-ground live biomass (AGB, dry weight) change. (**a**) Deviation of individual plot AGB change rate in absolute values (hereafter simply referred to as deviation) from expected mean plotted against monitoring length, showing negative trend. (**b**) Weighting all plots with cube root of monitoring length removed the trend. (**c**) No pattern when deviation was plotted against plot area. (**d**) Product of deviation and weight (cube root of monitoring length) plotted against weight showing no pattern, indicating the chosen weight is appropriate.

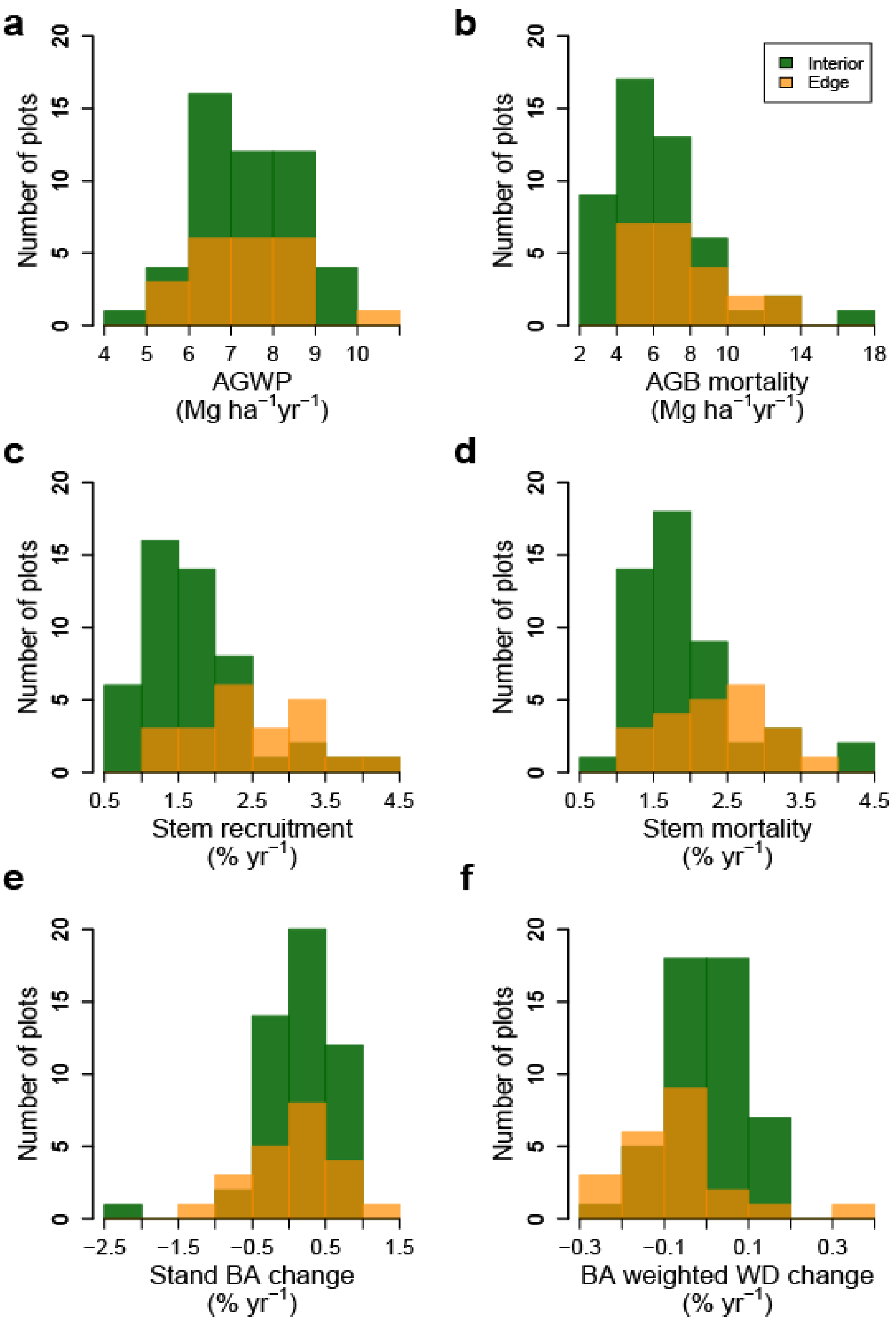

Supplementary Figure 5. Histograms showing variation in plot level long-term mean estimates of biomass dynamics in forest interior and edge affected plots. These include Above-ground wood productivity (AGWP, **a**), Above-ground live biomass (AGB) mortality (**b**), stem recruitment (**c**) and mortality (**d**) rates, and changes in stand basal area (BA, **e**) and BA weighted mean wood density (WD, **f**) calculated on a proportional basis relative to values of the initial censuses.

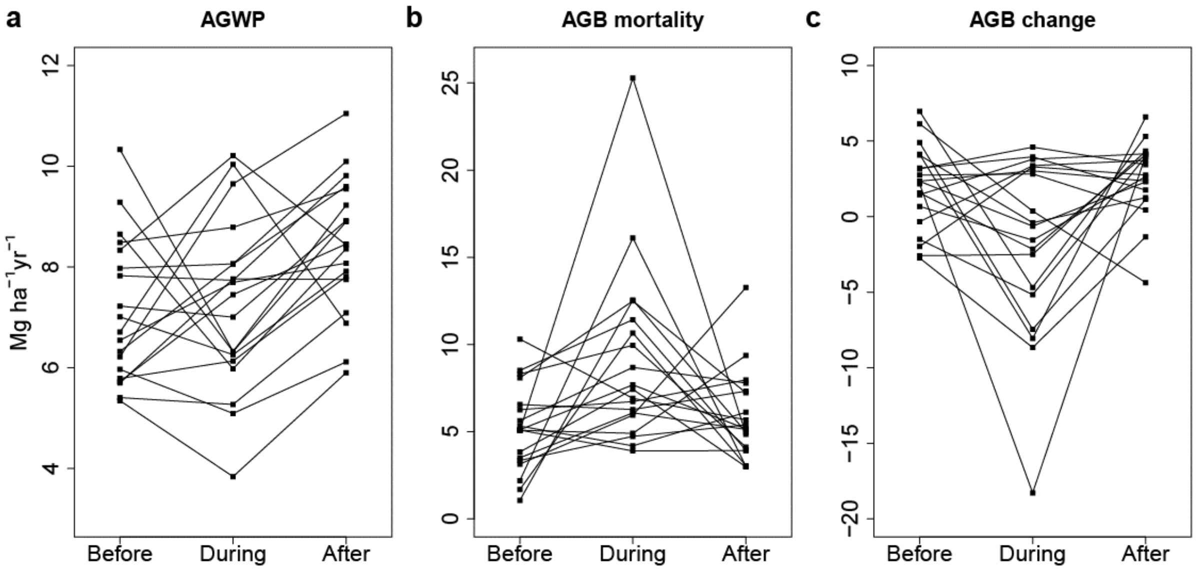

Supplementary Figure 6. Biomass dynamics in 19 forest interior plots in Borneo that were monitored over the 1997–98 El Niño. Symbols represent plot level means of above-ground wood productivity (AGWP, **a**), above-ground live biomass (AGB) mortality (**b**) and AGB change (**c**) calculated for three mean time intervals: before drought 1978.6–1996.5, during drought 1996.5–2000 and after drought 2000–2011.1. Lines represent individual plot trajectories over the three periods.

Supplementary Table 1. Exploring the impact of anthropogenic forest edge on biomass carbon dynamics in Borneo's forests and how these estimates were influenced by varying the threshold distance used to define edge. Variables shown are linear mixed effects (LME) model estimates of long-term above-ground live biomass (AGB) change (based on plot by census data), and above-ground wood productivity (AGWP) and AGB mortality (based on plot by census interval data), all in Mg ha^{-1} yr^{-1}. The 71 long-term forest monitoring plots were divided into 'edge' and 'interior' categories using incremental edge thresholds from 100 m, in 100 m steps to 1,000m, the lower (393 m) and upper (908 m) limits of the 95% CI and the estimated threshold (448 m) based on a hockey-stick model (see Methods). The results show that 'edge' and 'interior' plots behave differently, and that 'interior' plots consistently gained biomass, both largely irrespective of what distance is used to define edge. AGB change values in italics are significantly different from zero ($P < 0.05$). Values in bold font indicate the variable estimates differ significantly ($P < 0.05$) between edge and interior plots.

	Edge plots				Interior plots			
Threshold distance (m)	n	AGB change	AGWP	AGB mortality	n	AGB change	AGWP	AGB mortality
100	5	-1.09	7.75	8.99	66	*0.67*	7.37	6.61
200	10	***-1.35***	7.64	**9.84**	61	***0.87***	7.36	**6.39**
300	12	***-1.39***	7.58	**9.52**	59	***0.93***	7.36	**6.27**
393	17	***-1.06***	7.64	**8.28**	54	***1.06***	7.32	**6.18**
400	18	**-0.91**	7.54	**8.13**	53	***1.05***	7.34	**6.20**
448	22	**-0.28**	7.52	7.50	49	***0.91***	7.34	6.30
500	24	-0.10	7.37	7.02	47	*0.86*	7.39	6.41
600	28	0.02	7.29	6.91	43	*0.89*	7.44	6.50
700	30	0.13	7.32	6.90	41	*0.85*	7.43	6.39
800	31	0.14	7.32	6.94	40	*0.86*	7.43	6.34
900	35	0.31	7.30	6.75	36	*0.78*	7.46	6.45
908	35	0.31	7.30	6.75	36	*0.78*	7.46	6.45
1000	37	0.34	7.31	6.81	34	*0.78*	7.46	6.50

Supplementary Table 2. Parameters of the best local height-diameter (*H-D)* model for each of the forest types represented by the 71 long-term plots in this study. The mean differences in plot above-ground live biomass (AGB) based on local *H-D* model and the widely used Feldpausch equation were shown. Both local and Feldpausch equations are in the form of a Weibull function $H = a(1\text{-}\exp(-bD^c))$. The Feldpausch equation is parameterised with a = 57.122, b = 0.0332 and c = 0.8468. Mean AGB difference = AGB (Feldpausch *H-D*) – AGB (local *H-D*). Asterisks indicate significant difference from zero with $P < 0.05$.

Forest type	*a*	*b*	*c*	No. of plots for model fitting	No. of plots model applied to	Mean AGB difference from Feldpausch allometry (Mg ha^{-1})
Moist dipterocarp	107.670	0.0252	0.6772	38	53	7.32*
Wet dipterocarp	76.180	0.0493	0.6390	8	10	-13.99*
Moist kerangas	40.643	0.0675	0.7451	6	7	24.96*
Wet kerangas	74.737	0.0562	0.6514	1	1	-75.93

Supplementary Table 3. Model explanatory power of the linear mixed effects (LME) models of above-ground live biomass (AGB), stand basal area (BA) and BA weighted mean wood density (WD). Model explanatory power was assessed in terms of variability explained by fixed effects (marginal R-squared, R^2_m) and fixed and random effects combined (conditional R-squared, R^2_c). A common linear mixed effects (LME) model equation was employed to include *time*, *edge*, and *time x edge* interaction as fixed effects, treating plot identity as a random effect. Note that response variables vary more markedly in space (among plots) than in time (within plots), and model explanatory power is in terms of this combined spatio-temporal variation. The high R^2_c values indicate that the random effects captured most residual spatio-temporal variation not explained by the fixed effects.

Model response variable	R^2_m	R^2_c
AGB	0.11	1.00
plot mean BA	0.06	0.83
BA weighted plot mean WD	0.08	0.90
