# Supplementary material for: Long-term carbon sink in Borneo’s forests halted by drought and vulnerable to edge effects
Source: Nat Commun. 2017 Dec 19;8:1966. doi: 10.1038/s41467-017-01997-0 (PMC5736600; doi:10.1038/s41467-017-01997-0)
Supplement: Supplementary file 3 — Description of Additional Supplementary Files [file 41467_2017_1997_MOESM3_ESM.pdf]

Description of Additional Supplementary Files

File name: Supplementary Data 1

Description: Plot summary and metadata of 71 long-term forest monitoring plots in Borneo.
